# Fulfillment of administrative and professional obligations of hospitals and mission motivation of physicians

**DOI:** 10.1186/s12913-017-1990-0

**Published:** 2017-01-13

**Authors:** Jeroen Trybou, Paul Gemmel, Sebastian Desmidt, Lieven Annemans

**Affiliations:** Ghent University, Ghent, Belgium

**Keywords:** Mission Statement Motivation, Psychological Contract, Affective Organizational Commitment, Leader-Member exchange, Chief Medical Officer, Hospital-Physician Relationships

## Abstract

**Background:**

To be successful, hospitals must increasingly collaborate with their medical staff. One strategic tool that plays an important role is the mission statement of hospitals. The goal of this research was to study the relationship between the fulfillment of administrative and professional obligations of hospitals on physicians’ motivation to contribute to the mission of the hospital. Furthermore the mediating role of the physicians’ emotional attachment to the hospital and moderation effect of the exchange with the head physicians were considered.

**Methods:**

Self-employed physicians of six hospitals participated in a survey. Descriptive analyses and linear regression were used to analyse the data.

**Results:**

The results indicate that affective commitment mediated the relationship between psychological contract fulfillment and mission statement motivation. In addition, the quality of exchange with the Chief Medical Officer moderated the relationship between the fulfillment of administrative obligations and affective commitment positively.

**Conclusion:**

This study extends our understanding of social exchange processes and mission statement motivation of physicians. We showed that when physicians perceive a high level of fulfillment of their psychological contract they are more committed and more motivated to contribute to the mission statement. A high quality relationship between physician and Chief Medical Officer can enhance this reciprocity dynamic.

**Electronic supplementary material:**

The online version of this article (doi:10.1186/s12913-017-1990-0) contains supplementary material, which is available to authorized users.

## Background

Internationally, hospitals must rely on increased collaboration with their medical staff to organize high-quality care and deliver it efficiently within budgetary limits [1]. In the ever more complex and dynamic health care environment healthcare organizations search for strategic tools that enable them to improve organizational performance and motivate staff to perform at the highest possible level [2]. One strategic tool that has begun to play an increasingly important role in healthcare organizations is the mission statement [3]. This instrument is considered the cornerstone of every organizational strategy and is intended to capture the organization’s enduring purpose, practices and core values [4]. It has been argued that compared to other for-profit organizations the need for a mission statement is especially pronounced in the not-for-profit hospital sector. Moreover, the not-for-profit nature of hospitals implies that financial performance is not the critical performance measure. Rather, a hospital’s raison d’être is more broadly based. Specifically, it answers important questions about a hospital, such as, “Why do we exist?”, “What is our purpose?”, and “What do we want to achieve?” [5].

**Fig. 1:**
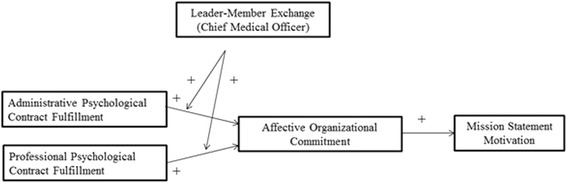
Study framework. This figure provides an overview of our study framework

However, the wide spread failure of realizing the mission statement is a problem that runs widely through the mission statement literature [6]. Accordingly, a central issue in contemporary research is how organizational members can be motivated to help realizing to the organization’s mission. In this paper we turn to the field of organizational behavior. More specifically we apply a social exchange perspective to study this aspect. Social exchange theory has gained prominence as the dominant framework for understanding the relationship between individual and organizations Over the past decades, both academics and practitioners have asserted a relationship between perceptions of high-quality social exchange and beneficial organizational attitudes, organizational behavior and organizational outcomes [7]. Specifically, to understand organizationally desired work attitudes and behaviors, scholars have often drawn on the construct of the psychological contract. This is defined as the individual beliefs, shaped by the organization, regarding terms of an exchange agreement between individual and organization [8]. Surprisingly, to our knowledge there have been no studies that applied the psychological contract to explain the degree of mission motivation. The objectives of this paper are to (a) develop a social exchange-based model of mission statement motivation, (b) to investigate the extent to which these relationships are mediated by affective organizational commitment as social exchange theory would predict and (c) to investigate the extent to which this relationship is moderated by physician senior leadership. Following a theoretical consideration of hospital-physician relationships, the mission statement, psychological contract and leader-member exchange, we introduce our hypotheses. The conceptual model guiding our study is outlined in Fig. 1. We then present an empirical field study with a sample of self-employed physician-specialists practicing at six Belgian hospitals designed to test our proposed model and hypotheses. We conclude the paper with a discussion of the implications of the findings.

### Hospital-Physician relationships

The relationship between hospital and physician is a significant research topic and a central concern of health policy makers and hospital executives. While these self-employed professionals are not a formal member of the organization they have a major impact on hospital performance [9]. Consequently increased cooperation between physician and hospital has become paramount. Internationally, hospitals have evolved from what was merely a physician workshop to an accountable organization delivering integrated care and health services [10]. However, while at first sight both hospital and physician seem to have the same goals (improve the health of patients), a closer look shows that the interests of the two parties overlap only partly and thus are not fully aligned [1]. Alignment refers to the degree to which physicians and hospitals share the same mission, vision, goals and objectives and work together toward their accomplishment [11].

Prior research has offered a number of important insights into alignment of the medical staff with the goals and objectives of the hospital [10]. We study the noneconomic relationship by applying the theoretical lens of social exchange (fulfillment of the physician’s psychological contract by the hospital and leader-member exchange) and complement this typology with a another aspect of alignment present in the management literature, mission statement motivation. Remarkably this view has seldom been linked to research on hospital-physician relationships.

### Organizational socialization and the mission statement

Organizational socialization refers to the process by which employees acquire knowledge about and adjust to organizational requirements in order to participate successfully as an organizational member [12]. A core element of organizational socialization is the organizational member’s knowledge and acceptance of organizational goals and values as this has significant impact on a person’s job and on members’ activities and attitudes [13]. In short, the more engaging, attractive and worth-while the organization’s goals and values are to people, the more they will support them [14]. In order to capitalize on the motivational power of their goals and values, organizations should generate a shared sense of vision, mission, and purpose among organizational members, providing confidence and direction about the future of the organization. The appeal to broader goals activates the higher-order needs of individuals, encouraging them to transcend their own self-interest for the sake of the organization and its customers [15].

In the last decades, an increasing number of organizations, including hospitals, have turned to clarify the organization’s purpose by articulating a mission statement. As a formal written document intended to capture an organization’s unique and enduring purpose, practices, and core values, the mission statement is considered to be critical to the organizational success of hospitals and the starting point of virtually every strategic management initiative [4]. Moreover, one of the primary goals of mission statements is to create a shared sense of organizational meaning by projecting a specific description of the organization’s essence. The central aim of the mission statement is to express what is (un)important depending on shared interests and underlying values [16]. By clarifying their purpose and social contribution, organizations hope to motivate and inspire organizational members to support the organization in its pursuit to realize the organizational rationale [5]. The construction of such a shared organizational definition stimulates the functioning of the organization as it provides clear directions and goals to organizational members and increases their sense of connection with the organization [17].

In case of physicians this view is considered highly relevant and contributes to the literature. Moreover, previous studies have been criticized because they apply a unilateral economic view by assuming that physician’s motivation is primarily based on self-interest and thereby the professional context and social relationships is ignored. These studies do not acknowledge that physicians, as professionals, have a more complex set of motives that steers their behavior and intrinsic rewards supported by hospitals could therefore contribute to alignment [18]. We argue that mission statements can help building effective hospital-physician relationships. Fundamental alignment of values and purpose is necessary to enable a satisfactory (non-financial) ‘buy-in’ among physicians. In addition, it has been asserted that noneconomic aspects, aiming at improving the hospital work environment and increasing the added value of the hospital to the members of medical staff, are critically important to physician-hospital alignment [10]. We study the noneconomic relationship through the theoretical lens of social exchange and argue that noneconomic aspects reinforce mission statement motivation.

### Social exchange theory and the psychological contract

Social exchange theory is considered one of the dominant framework for understanding organizational behavior [7]. According to this theory, organizational members tend to reciprocate the beneficial (detrimental) treatment they perceive with positive (negative) work-related behavior and attitudes [19, 20]. In this respect, perceptions of physicians about the relationship with the hospital could increase hospitals’ ability to motivate physicians. Scholars have often drawn on social exchange-based constructs to explain a variety of work attitudes (e.g. organizational commitment) and behaviour (e.g. extra-role behaviour), resulting in a body of empirical evidence in support of the nom of reciprocity principle [21]. The psychological contract refers to the beliefs of individuals with respect to the conditions and terms of the exchange agreement with their organization. It refers to the way the working relationship is interpreted and understood by individuals. These beliefs are rooted in the idea that both explicit as well as implicit promises have been made in exchange of services that are offered, installing a set of reciprocal obligations [22].

Importantly It has been shown that in the case of physicians ideological pluralism is present within the psychological contract. Specifically within the psychological contract of physicians a distinction can be made between a professional and an administrative dimension. This is induced by differences between models of organizing that are based on administrative/organizational (management) principles and those models that are based on professional and occupational organizing principles of medicine which converge in a hospital setting [23, 24]. The psychological contract therefore involves both professional and administrative roles and perceived obligations. It have been shown that both roles are relevant to understand how physicians relate to the hospital they practice at. In other words, physicians interact with the hospital both as an organizational member and as a professional. As an organizational member, they ascribe particular roles (a set of perceived rights and obligations) to the organization that relate to the ideology of the administrative organization. As a professional, they ascribe particular roles to the organization that relate to the ideology of professional work. Consequently, a professional’s response could differ depending on whether professional or administrative role obligations are fulfilled.

While an extensive body of empirical evidence in support of the norm of reciprocity with respect to a variety of attitudinal and behavioral organizational outcomes exists previous studies have not focused on the concept of mission statement motivation. This concept indicates to what extent organizational members are motivated to contribute to the achievement of the organizations’ mission. It focuses on the extension of effort toward achieving it [25].

Hypothesis 1a: Administrative psychological contract fulfillment is positively associated with mission statement motivation.

Hypothesis 1b: Professional psychological contract fulfillment is positively associated with mission statement motivation.

### Affective commitment

As argued by Blau [20] repeated social exchange gestures of an individual depend on the evaluation of the relationship. This explains why affective organizational commitment has been found to be related to positive organizational behaviors such as organizational citizenship [26] and suggests that it is potentially to other forms of organizational behavior such as mission statement motivation. Affective organizational commitment is an individual’s emotional attachment to, involvement in, and identification with an organization [27]. Moreover the concept of affective commitment is implicitly present in Blau’s [20] conceptualization of social exchange from which many scholars have drawn their theoretical rationale for a relationship between the fulfillment of the psychological contract and organizationally desired behavior. Because of this solid conceptual foundation organizational commitment is one of the most widely studied attitudinal antecedents of organizational behavior [28]. We therefore include affective commitment as a possible mediator in our model. Specifically, drawing on the reciprocity principle affective commitment is an intermediate outcome of (administrative and professional) psychological contract fulfillment and leads to organizationally desired behavior (mission statement motivation).

Hypothesis 2: Affective commitment mediates the relationship between psychological contract fulfillment and mission statement motivation.

### Leader-Member exchange

In general it has been shown that leaders and managers within organizations have an important impact on the relationship between individual and organization. Moreover he or she is a representative of the organization and a purveyor of resources and support [29]. Previous studies did not investigate how physician-hospital exchange interrelates with physician senior leadership. Specifically, in hospitals the Chief Medical Officer (CMO) is responsible for governing physician-hospital relationships and shaping physician-hospital exchanges. One theory that studies leaders’ influence on organizational members is Leader-Member eXchange (LMX). This social exchange-based theory poses that leaders form different relationships with organizational members and high-quality LMX leads to organizational behaviour and attitudes that are beneficial to the organization [30]. We therefore expect that LMX has an effect on the reciprocity principle. More precisely, we address the perceived quality of exchange with the CMO, as a potential moderator of the relationship between perceptions of psychological contract fulfillment and affective organizational commitment.

Hypothesis 3: LMX CMO positively moderates the relationship of psychological contract fulfillment and affective commitment such that this relationship is stronger when LMX is high.

## Methods

### Sample

Six Belgian hospitals (Flanders region) participated. Seven hundred sixty one physicians were surveyed. In total, 130 physicians participated (17.1% response rate).

### Study setting

In Belgium, physician-specialists praMictice prevailingly as liberal, self-employed professionals. From a reimbursement point of view, physicians have a distinct revenue stream. The hospital is reimbursed for the operating expenses by a DRG-based hospital budget. This budget covers the cost of nursing, pharmaceuticals, the hotel costs etc. The physician is separately reimbursed by means of a professional fee for his/her activities.

### Measures

The survey was composed of previously validated instruments which previously demonstrated sound psychometric properties. Questions were forward-translated into Dutch and back-translated as a check to assure an adequate questionnaire. All measures were assessed using a five-point Likert-type scale (1 = strongly disagree; 2 = disagree; 3 = neither agree nor disagree; 4 = agree; and 5 = strongly agree). A full overview of the survey is given in Additional file 1.

#### Psychological contract fulfillment

Different instruments to study the psychological contract have been developed. Since we focus on a particular type of content—namely the difference between organizational and professional aspects—we employed the scale of Bunderson et al. [30]. Six items referring to organizational obligations and six items referring to professional obligations were used. We used the principal components method and omitted four items that loaded less than 0.6 on their own factor, or more than 0.4, resulting in a final eight items (four items referring to organizational aspects and four to professional aspects). Examples of items are ‘business oriented’ and ‘fosters quality of care’. The Cronbach’s alpha reliability coefficients were satisfactory (α = 0.85 for administrative breach and α = 0.83 for professional breach).

#### Mediating variable

Affective organizational commitment was measured with the five items of the Allen and Meyer scale [27]. A sample question is ‘I consider the hospital’s problems as may own problems’. Cronbach’s alpha was 0.85.

#### Dependent variable

Mission statement motivation was measured by a 3-item measure based on the scale of Desmidt & Prinzie [25] which has demonstrated adequate levels of reliability and construct validity. A sample item is ‘I support the goals this organization strives for’. The Cronbach’s alpha was acceptable (α = 0.79).

#### Moderating variables

The seven-measure of LMX concentrated on the relationship between physician and Chief Medical Officer and was based on the measure developed by Scandura and Graen [31]. An exemplary question is ‘What are the chances that the CMO would be inclined to use power to help you solve problems in your work?’ The Cronbach’s alpha in our study was α = 0.90.

#### Control variables

Following previous studies (e.g. [32]) we controlled for tenure and gender.

#### Ethical considerations

The study was approved by the medical ethics committee of Ghent University and the University Hospital Ghent.

#### Analyses

We first examined the distinctiveness of the variables included in this study using confirmatory factor analysis and the maximum likelihood method of estimation. The hypothesized model including five factors (i.e. administrative and professional psychological contract fulfillment, leader-member exchange, organizational commitment and mission statement motivation) was compared to a series of more parsimonious models. The differences between these models was examined using χ^2^difference test [33]. In addition to the χ^2^test, the following fit indices were used: the incremental fit index, the comparative fit index and the root mean square error of approximation. Table 1 provides an overview of our results. Our hypothesized five-factor model yielded a good fit to the data and outperformed the parsimonious models. These results demonstrate the distinctiveness of the study’s variables.

**Table 1:** Factor analyses

Model	χ^2^	*df*	∆ χ^2^	∆ *df*	IFI	CFI	RMSEA
Hypothesized 5-factor model	323,52	218	-	-	.95	.95	.061
Combining professional and administrative fulfillment	396,22	222	72,7***	4	.91	.91	.08
Combining professional fulfillment and affective commitment	514,8	222	191,28***	4	.85	.85	.101
Combining administrative fulfillment and affective commitment	442,89	222	119,37***	4	.89	.89	.088
Combining mission motivation and affective commitment	43.84	222	107,32***	4	.89	.89	.085
Combining professional fulfillment and mission motivation	488,33	222	164,81***	4	.86	.86	.096
Combining administrative fulfillment and mission motivation	411,24	222	87,72***	4	.9	.9	.081
Combining professional fulfillment and LMX	513,76	222	19.24***	4	.85	.85	.101
Combining administrative fulfillment and LMX	469,2	222	145,68***	4	.87	.087	.093
Combining affective commitment and LMX	542,69	222	219,17***	4	.84	.83	.106
Combining mission motivation and LMX	525,55	222	202,03***	4	.85	.84	.103
One-factor model	97.2	228	646,68***	10	.52	.62	.159

***p < .001

The Statistical Package for Social Sciences was used (SPSS 20.0 for Windows, Inc., Chicago, IL, USA). Descriptive statistics were used to analyze demographic variables. Correlation analysis was run to test the correlations between all independent, dependent and mediator and moderator variables.

Baron and Kenny’s [34] path analysis procedure was used for testing mediating variables. The following steps were applied. In the first and second step, the independent variable (psychological contract fulfillment) should be significantly correlated with the dependent variable (mission statement motivation) and mediator (affective organizational commitment). In the third step, the mediator (affective commitment) must be correlated with the dependent variable (mission statement motivation). In the fourth step, the independent variable (psychological contract fulfillment) must no longer be correlated with the dependent variable (mission statement motivation) when the mediating variable (affective commitment) is also included in the regression equation.

To test the moderating influence of LMX, we conducted moderated regression analysis. To avoid multicollinearity, we first centered the independent variable and moderator. Thereafter these centered variables were multiplied and entered while controlling for their main effects.

## Results

### Demographic characteristics

The characteristics of the sample are shown in Table 2. On average the respondents were 47 years old and 60.4% had more than 10 years of experience in the same hospital. The majority of the respondents were male (61.5%) and 30% were surgeons.

**Table 2:** Sample demographics and personal factors

Personal factor	Subcategory	Mean (SD) or percentage
Age	-	47 (9.14)
Gender	Male	61.5
	Female	38.5
Tenure	<5 years	23.1
	5–10 years	24.3
	11–15 years	13.0
	16–20 years	16.6
	>20 years	23.1

Through correlation analyses we determined whether there were differences in psychological contract fulfillment, affective commitment, leader-member exchange and mission statement motivation in terms of gender and tenure. No personal characteristics were significantly related (Spearman correlation coefficient). Table 3 provides an overview.

**Table 3:** Correlation analyses

Variable	M	SD	1	2	3	4	5	6
1. Gender	-	-	-					
2. Tenure	-	-	-.187*	-				
3. Administrative Psychological Contract Fulfillment	2.25	.71	.104	.048	-			
4. Professional Psychological Contract Fulfillment	2.25	.73	.119	.110	.557**	-		
5. Leader-Member Exchange	3.00	1.01	.170	.038	.446**	.452**	-	
6. Affective Organizational Commitment	3.60	.82	-.003	.104	.361**	.221*	.356**	-
7. Mission Statement Motivation	3.45	.82	.126	.024	.511**	.405**	.403**	.486**

*p < .05, **p < .01

### Psychological contract fulfillment and mission statement motivation

As shown in Table 3, the results show that administrative (*r* = 0.361, *p* < 0.01) and professional (*r* = 0.221, *p* < 0.01) psychological contract fulfillment and affective commitment, affective commitment and mission statement motivation (*r* = 0.486 *p* < 0.01) and administrative (*r* = 0.511, *p* < 0.01) and professional (*r* = 0.405, *p* < 0.01) psychological contract fulfillment and mission statement motivation are related. This means that physicians who perceived higher levels of psychological fulfillment are more strongly commited to the hospital and more motivated to contribute to the realization of the hospital’s mission statement.

Furthermore our results demonstrate a relationship between LMX and administrative (*r* = 0.446, *p* < 0.01) and professional (*r* = 0.452, *p* < 0.01), psychological contract fulfillment, LMX and affective commitment (*r* = 0.356, *p* < 0.01) and LMX and mission statement motivation (*r* = 0.403, *p* < 0.01). This means that physicians who perceived higher quality of exchange are more commited to the hospital and more motivated to contribute to the hospital’s mission statement.

### Moderating effects of LMX

**Fig. 2:**
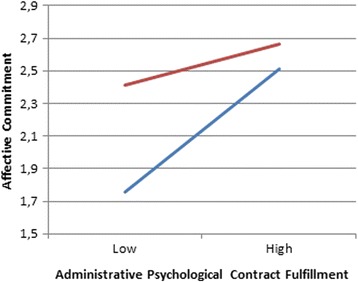
Moderating effect of LMX CMO. This figure illustrates the moderating effect of quality of exchange with the CMO on the positive relationship between administrative psychological contract fulfillment and mission statement motivation. The blue line is used to illustrate low LMX. The red line is used to illustrate high LMX

Moderated multiple regression was used to examine the moderating hypotheses. Gender and tenure were entered at step 1 as control variables. Our main predictors (all centered) were entered at step 2. Finally, the product terms of interest for this study were entered at step 3. The results are presented in Table 4. Administrative psychological contract fulfillment X leader-member exchange was a significant predictor of affective commitment (β = −0.146, *t* = −2,062, *p* = 0.041). To understand the form of this interaction we plotted the regression for affective commitment on administrative psychological contract fulfillment respectively at 1 SD above the mean of LMX. Figure 2 illustrates that a high quality of exchange with the CMO has a positive effect on the positive relationship between administrative psychological contract fulfillment and mission statement motivation. The slopes of the regression lines differ from each other and therefore illustrate a buffering effect of the CMO. This result implies that physicians with high quality of exchange with the CMO are less sensitive to perceptions of the (un)fulfillment of the administrative psychological contract;

**Table 4:** Results of moderated regression for affective commitment

		Model 1		Model 2		Model 2	
Step	Variables entered	β	ΔR^2^	β	ΔR^2^	β	ΔR^2^
1	Gender	-.063		-.08		-.069	
	Tenure	.085		.107		.087	
			.011		.011		.011
2	Administrative Psychological Contract Fulfilment	.279**		.306**		.273**	
	Professional Psychological Contract Fulfilment	-.055		-.078		-.047	
	Leader-Member Exchange Chief Medical Officer	.264**		.248**		.266**	
			.181***		.181		.181
3	APCF X Leader-Member Exchange			-.169*			
					.027***		
	PPCF X Leader-Member Exchange					-.044	
							.002***

Note. *N* = 130. Standardized regression coefficients are reported

^+^ *p* < .10, *p < .05, **p<,01, ***p < .001

Professional psychological contract fulfillment X leader-member exchange was not a significant predictor of affective commitment (β = −0.044, *t* = −0.529, *p* = 0.598). In contrast to our finding when considering administrative psychological contract we can therefore not identify a buffering effect of the CMO with respect to the fulfillment of the professional psychological contract and mission statement motivation.

Blue line: low LMX – Red line: high LMX

### Mediation effect of affective commitment

Before testing our mediation hypotheses we ran a multiple regression analysis using mission statement motivation as the dependent variable. Demographics were entered as controls at step 1, psychological contract variables and leader-members exchange (all centered) were entered at step 2, the interaction terms at step 3 and affective commitment at step 4. Table 5 provides an overview. Table 5 shows that the administrative psyXchological contract fulfillment leader-member exchange (β = 0.194, *p* = 0.0351) and affective commitment (β = 0.288, *p* < 0.001) were associated with mission statement motivation in the final model demonstrating mediation.

**Table 5:** Results of Multiple Regression for Organizational Citizenship Behavior

		Model 1		Model 2		Model 3		Model 4		Model 5	
Step	Variables entered	β	ΔR^2^	β	ΔR^2^	β	ΔR^2^	β	ΔR^2^	β	ΔR^2^
1	Gender	.131		.059		.039		.012		.031	
	Tenure	.039		-.001		-.004		.031		-.002	
			.002								
2	Administrative Psychological Contract Fulfillment			.411***		.361***		.398***		.305***	
	Professional Psychological Contract Fulfillment			.166^+^		.114		.082		.111	
					.259***						
3	Leader-Member Exchange Chief Medical Officer					.185*		.161^+^		.093	
							.021***				
4	APCF X Leader-Member Exchange							-.260**		-.194*	
	PPCF X Leader-Member Exchange							-.016		-.042	
									.06***		
5	Affective Organizational Commitment									.288***	
											.63***

Note. *N* = 130. Standardized regression coefficients are reported.

*APCF* administrative psychological contract fulfillment, *PPCF* professional psychological contract fulfillment

^+^ *p* < .10, *p < .05, **p < .,01, ***p < .001

## Discussion

This study is among the few to study social exchange in relation to the concept of mission statement motivation. We contribute to the existing body of knowledge by focusing on the importance of administrative and professional psychological contract fulfillment and the role of affective organizational commitment and physician leadership (leader-member exchange) in the reciprocity dynamic.

We set out to understand better how self-employed physicians’ reciprocity behavior in social exchange with hospitals in terms of their mission statement motivation and how this is influenced by their relationship with the chief medical officer. The outcomes of this study support the proposed conceptual model. First, our results showed a relationship between both administrative and professional psychological contract fulfillment and mission statement motivation. This relationship was mediated by affective organizational commitment.

This finding extends our knowledge of social exchange processes. While there is a large body of evidence supporting the importance of the psychological contract to several organizational attitudes and behavior [35], insight into the interconnection of these concepts remains a challenge [36]. Furthermore the link between quality of social exchange and the concepts of mission statement have been absent in the literature. We contribute to the literature (i) by demonstrating the significance of both administrative and professional dimensions of physician-hospital exchange and (i) with evidence on affective commitment as an immediate driver of mission statement motivation. In addition we add to the literature by studying the psychological contract in a sample of self-employed physicians.

Second, we found that when physicians had high levels of leader-member exchange with the chief medical officer they adhered more strongly to the norm of positive reciprocity with respect to the fulfillment of administrative obligations. This finding illustrates the importance of physician senior leadership to hospital-physician relationships. Our study advances previous research by showing how professional-organization exchange is more complex than previously assumed. However, our study did not find a significant moderating impact with respect to professional psychological contract breach. Given the professional role of the CMO in shaping the professional work setting of physicians in hospitals and governing the medical-professional relationship this is surprising. The effects of pphysician senior leadership needs further study. Previous studies demonstrated the important role for physician-executives with respect to – sometimes conflicting - managerial and clinical issues [37]. However our results show that physicians’ commitment is only influenced by the CMO-exchange when administrative issues and not professional issues raise. A possible explanation for this finding is that by gaining credibility as managers, CMO’s may lose their credibility as a medical doctor [38]. The CMO is viewed as a member of the executive team primarily dealing with administrative issues and not a ‘physician’ concentrating on professional medical issues.

### Practical implications

Mission statements are considered the cornerstone of organizational strategy, the need for a mission statement Xis especially pronounced in hospital-physician relationships. First, the not-for-profit nature of hospitals implies that the organizational goal is more broadly based compared to the traditional for-profit companies. Second, physicians are professionals and have a complex set of motives of behaviour. While formulating mission statements is difficult, executing or implementing it throughout the organization is even more difficult [39]. Moreover, the wide spread failure of mission statements is a consistent theme that runs through the mission statement literature [40]. This is particularly the case when alignment of physicians is considered. Relationships with these professionals have been described as strained and lukewarm at best [18]. Although they are mostly self-employed and thus not formal members of the organization, physicians have a major impact on hospital performance [1].

Drawing on social exchange theory, we demonstrate that by improving the perceived quality of exchange physician-hospital alignment can be improved. Specifically, when physicians perceive that hospitals’ professional and administrative obligations are fulfilled they are more motivated to contribute to the hospital’s mission.

### Limitations

Despite the value of this study several limitations should be considered. First all six hospitals were situated in Belgium. Since hospital-physician relationships differ between regions and countries (e.g. because of the regulatory framework and market conditions) the results may not be generalized to easily to other regions. More precisely we did study the specific Belgian setting in which physician-specialists practice prevailingly as liberal, self-employed professionals. It would be valuable to investigate how physician employment contracts impact psychological contract fulfillment and mission statement motivation compared to self-employed physicians. In addition the number of participants and our response rate was limited. This observation is similar to previous studies of physicians [41]. We compared respondents to non-respondents in terms of gender, tenure and specialty (surgeon vs. non-surgeon). With respect to these characteristics no differences were present. However it may be that physicians who did not respond may have reported different results which could have an impact on our results. Furthermore the cross-sectional design does not permit to demonstrate causality. Finally the respondents of this study provided information on the independent variables, mediators, and dependent variables. Although we limited the risk of common-method bias by applying the guidelines of Podsakoff et al. [41] and performing a Harmon’s single factor test the potential for common-method exists. Finally, our study did not include hospital outcomes. The investigation of the impact of mission statement motivation and executive-physician relationships on hospital performance should be considered as an avenue for future research.

## Conclusion

This study examined the effect of affective organizational commitment and leader-member exchange in the relationship of administrative and professional psychological contract fulfillment and mission statement motivation among self-employed physicians. Our findings show that affective organizational commitment mediates this relationship. Furthermore high-quality leader-member exchange between chief medical officer and physician staff enhances the effects of fulfilled administrative obligations to physicians’ emotional attachment to the hospital and thereby mission statement motivation. We believe that future research should further explore how organizational members can be motivated to help realize the mission.

## Additional file

Additional file 1: Survey provides an overview of the questions included in the survey. (DOCX 17 kb)

## Abbreviations

CMO: Chief Medical Officer
LMX: Leader-Member Exchange
