# Supplementary material for: Fulfillment of administrative and professional obligations of hospitals and mission motivation of physicians
Source: BMC Health Serv Res. 2017 Jan 13;17:28. doi: 10.1186/s12913-017-1990-0 (PMC5237190; doi:10.1186/s12913-017-1990-0)
Supplement: Additional file 1: — Survey provides an overview of the questions included in the survey. (DOCX 17 kb) [file 12913_2017_1990_MOESM1_ESM.docx]

**Additional file: Measurement Instruments**

**Psychological Contract**

(Based on Bunderson et al., 2000)

| The following table shows a number of possible characteristics of the hospital. To what extent does the hospital fulfill each of the following expectations? (score 1-5) | | | | |
| --- | --- | --- | --- | --- |
|  |  |  |  |  |
| 1. An integrated functioning | | | | |
| 1. Striving for hospital-wide coordination | | | | |
| 1. Emphasizing hospital-wide goals | | | | |
| 1. A competent medical staff | | | | |
| 1. Stimulating clinical excellence | | | | |
| 1. Stresses high quality care | | | | |
| 1. External proactive and entrepreneurial | | | | |
| 1. Business oriented | | | | |
| 1. Competitive with other hospitals | | | | |
| 1. Aligned with the hospitals in the area | | | | |
| 1. Financial access for patients is preserved | | | | |
| 1. Justified use of public resources | | | | |

**Quality of Leader-Member Exchange (Chief Medical Officer) (score 1-5)**

(Based on Scandura & Graen, 1984)

| 1. Do you usually feel that you know where you stand … do you usually know the head physician’s (HP) viewpoint? |
| --- |
| 1. How well do you feel that the HP understands your problems and needs as a physician in the hospital? |
| 1. How well do you feel that the HP recognizes your potential? |
| 1. What are the chances that the HP would be inclined to use power to help you solve problems in your work? |
| 1. To what extent can you count on the HP to bail you out at his or her expense when you really need it? |
| 1. I have enough confidence in the HP that I would defend and justify his or her decisions if he was not present to do so. |
| 1. How would you characterize your professional working relationship with the HP? |

**Affective Organizational Commitment (score 1-5)**

(Based on Allen & Meyer, 1990)

| 1. I consider the hospital's problems as my own problems |
| --- |
| 1. I am emotionally attached to this hospital |
| 1. This hospital means a lot to me |
| 1. I feel at home in this hospital |
| 1. I feel like 'a member of the family' in this hospital |

**Mission Statement Motivation (score 1-5)**

(Based on Desmidt & Prinzie, 2009)

1. I subscribe to my hospital’s vision
2. I support the goals this hospital strives for
3. I do not agree with what the organization maintains
